# Prognostic role of systemic immune-inflammation index in solid tumors: a systematic review and meta-analysis

**DOI:** 10.18632/oncotarget.18856

**Published:** 2017-06-29

**Authors:** Jie-Hui Zhong, Dan-Hui Huang, Zi-Yu Chen

**Affiliations:** ^1^ Department of Clinical Medicine, The First Clinical Medical College, Southern Medical University, Guangzhou, 510515, China

**Keywords:** systemic immune-inflammation index, solid tumors, meta-analysis

## Abstract

**Background:**

Inflammation may play an important role in cancer progression, and a higher systemic immune-inflammation index (SII) has been reported to be a poor prognostic marker in several malignancies. However, the results of published studies are inconsistent.

**Materials and Methods:**

A systematic review of databases was conducted to search for publications regarding the association between blood SII and clinical outcome in solid tumors with a date up to February 12, 2017. The primary outcome was overall survival (OS) and the secondary outcomes were progression-free survival (PFS) and cancer-specific survival (CSS). Pooled hazard ratios (HRs) and 95% confidence intervals (CIs) were used to assess the strength of the association between blood SII and clinical outcome in solid tumors.

**Results:**

A total of 15 articles were included in the analysis. Overall, systemic immune-inflammation index greater than the cutoff predicted poor overall survival (HR = 1.55, 95% CI = 1.27–1.88; *P* < 0.001). Subgroup analyses revealed that high systemic immune-inflammation index indicated a worse overall survival in hepatocellular carcinoma (*P* < 0.001), urinary cancers (*P* < 0.001), gastrointestinal tract cancers (*P =* 0.02), small cell lung cancer (*P* < 0.05) and acral melanoma (*P* < 0.001). Hazard ratio for systemic immune-inflammation index greater than the cutoff for cancer-specific survival was 1.44 (*P* < 0.05).

**Conclusions:**

Elevated systemic immune-inflammation index is associated with a worse overall survival in many solid tumors. The systemic-inflammation index can act as a powerful prognostic indicator of poor outcome in patients with solid tumors.

## INTRODUCTION

Inflammatory reaction play crucial role in shaping tumor development in many aspects, ranging from tumor initiation to tumor metastasis [1]. Inflammatory related peripheral cells (neutrophils, lymphocytes and platelets) derived from the peripheral blood were significantly associated with tumor progression in various tumors [2–4]. Moreover, infiammatory indexes (II) obtained with different combinations of these factors, such as neutrophil to lymphocyte ratio (NLR) and platelet to lymphocyte ratio (PLR), have been investigated as useful prognostic factors in various malignant solid tumors [5–7]. A novel inflammatory index, the systemic immune inflammation index (SII), defined as follows: SII = P*N/L, where P, N and L were the peripheral platelet, neutrophil and lymphocyte counts [8], was recently investigated as a prognostic marker in various malignancies. Geng et al. [9] suggested that SII was superior to the other systemic inflammation index such as PLR and NLR, and served as a more objective marker that reflects the balance between host inflammatory and immune response status.

The goal of the present study was to conduct a meta-analysis to investigate the association between peripheral blood SII in solid tumors and clinical outcome. Our hypothesis was that elevated SII correlates with worse OS and may thus serve as a readily available and inexpensive prognostic marker in clinical practice.

## MATERIALS AND METHODS

### Study identification and selection

Studies regarding the association between SII and clinical outcome in solid tumors published before February 12, 2017 were included through PubMed, Embase and Cochrane library searching by using the following terms and key words: (systemic immune inflammation index OR SII) AND cancer. The criteria used for the study selection were as followed: 1) studies were concerned about the prognostic impact of SII; 2) A hazard ratio (HR) and 95% confidence interval (CI) for overall survival (OS), cancer-specific survival (CSS) and progression-free survival (PFS) was available; 3) there were no overlapping data. Two of the authors (Jiehui Zhong, Danhui Huang) evaluated the eligibility of all studies independently collected from the databases based on the selection criteria. The Newcastle-Ottawa Scale (NOS) was used to assess study quality, which consists of three parameters of quality: selection (0–4 points), comparability (0–2 points), and outcome assessment (0–3 points) (Supplementary Table 1). Studies with the scores ≥ 6 were assigned as high-quality studies.

### Data extraction

Information was carefully extracted from all the eligible studies independently by three investigators according to the selection criteria listed above. The following data were collected: first author’s name, publication year, country, type of publication (abstract, full text), number of patients included in analysis, disease site, disease stage (metastatic, nonmetastatic, mixed [metastatic and nonmetastatic]), collection of data (prospective, retrospective), cutoff defining high SII used for peripheral blood SII, and hazard ratios and 95% confidence interval (CI) for OS, CSS, or RFS as applicable. Hazard ratios based on multivariable analyses other than univariate analyses were extracted prior. We did not require a minimum number of patients to be included in our meta-analysis.

### Statistical analysis

Meta-analysis was conducted using RevMan 5.3 analysis software (Cochrane Collaboration, Copenhagen, Denmark). Estimation of hazard ratios was pooled using the random-effect model or the fixed-effect model. Heterogeneity assumption was tested by a chi-square-based *Q* test. Proportion of the total variation across studies due to heterogeneity was checked by *I*-square statistics [10]. A *P*-value of > 0.05 for the *Q*-test indicated a lack of heterogeneity among studies, so that the pooled HR estimate of each study was calculated by the fixed-effect model [11]. Otherwise, the random-effect model was used [12]. Subgroup analyses were conducted according to disease site (i.e., hepatocellular carcinoma or urinary cancers), disease stage (metastatic/mixed or nonmetastatic), cutoffs for SII (SII ≥ 572 or SII < 572), data collection (prospective or retrospective), analysis of HR (multivariable or univariable) and article type (abstract or full paper). Differences between the subgroups were assessed using methods described by Deeks et al. [13]. The effect of SII cutoff on the hazard ratio for OS was evaluated by meta-regression analysis. Publication bias is the tendency on the parts of investigators, reviewers, and editors to submit or accept manuscripts for publication based on the direction or strength of the study findings [14]. An estimate of potential publication bias was carried out by the funnel plot, in which the standard error of log (HR) of each study was plotted against its log (HR). Funnel plot asymmetry was further assessed by the method of Egger’s linear regression test (*P <* 0.05 was considered a significant publication bias) [15].

## RESULTS

### Extraction process and study characteristics

**Figure 1:**
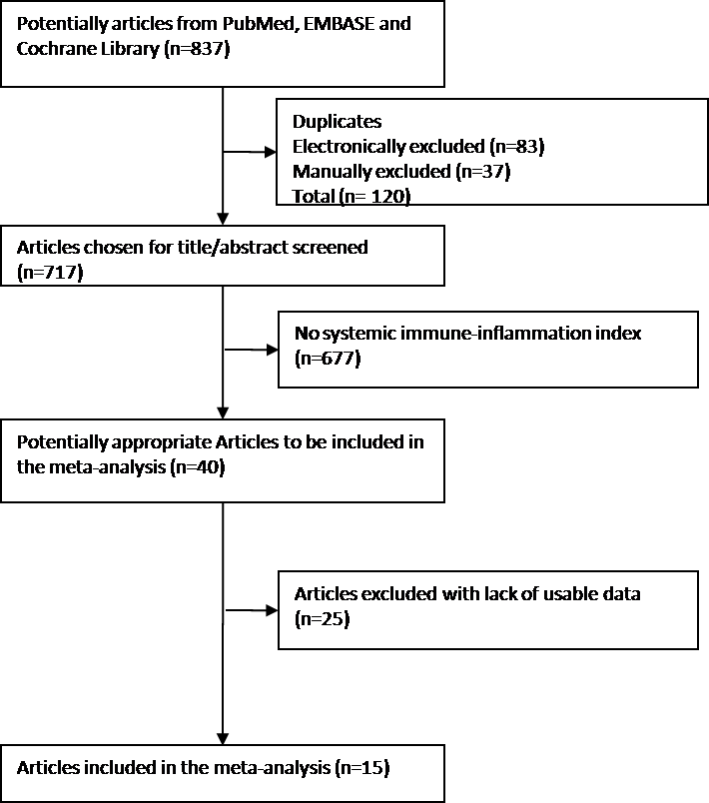
Flow diagram of included studies for this meta-analysis

The selection procedure was listed in Figure 1. Totally, 837 articles of interest were found after preliminary search, and 40 of them have relevant content on the association between SII and clinical outcome in solid tumors. Among them, 25 studies met the exclusion criterion due to duplication or lack of available data. Hence, 15 publications [8, 9, 16–28] including 16 studies (4875 patients) were selected. The characteristics of the studies included were shown in Table 1. Of them, there were 5 hepatocellular carcinoma studies, 2 esophageal squamous cell carcinoma studies, 2 gastric cancer studies, 1 renal cell cancer study, 1 malignant obstructive jaundice study, 1 small cell lung cancer studies, 1 colorectal cancer study, 1 biliary tract cancer study, and 1 metastatic castration-resistant prostate cancer study and 1 acral melanoma study. The quality of all 16 studies ranged from 5 to 7 (Supplementary Table 1), indicating that most studies included were high quality.

**Table 1:** Characteristics of studies included in the meta-analysis

Author	Study Period	Data collection	Ethnicity	Stage	Disease site	Number	Cut-off	Reported endpoints	Follow-up Median (month)
Hu 2014 1	2005–2006	retrospective	China	mixed	HCC	133	330	OS,	61.3
Hu 2014 2	2010–2011	retrospective	China	mixed	HCC	123	330	OS	28.8
Lolli 2016 1	2006–2014	retrospective	Italy	metastatic	RCC	335	730	OS, PFS	49
Lolli 2016 2	2011–2015	retrospective	Italy	metastatic	mCRPC	230	535	OS	29
Yang 2015	2009–2015	retrospective	China	mixed	HCC	189	300	OS	19.8
Passardic 2016	2007–2012	prospective	Italy	metastatic	CRC	289	730	OS, PFS	36
Feng 2016	2005–2008	retrospective	China	nonmetastatic	ESCC	298	410	CSS	NR
Ha 2016	2004–2009	retrospective	Korea	NR	BTC	158	572.38	OS	95.3
Geng 2016	2002–2012	retrospective	China	nonmetastatic	ESCC	916	307	OS	39
Jin 2016	2012–2016	retrospective	China	NR	MOJ	33	644	OS	10
Hong 2015	2000–2012	retrospective	China	mixed	SCLC	919	1600	OS	NR
Liu 2015	2005–2010	retrospective	China	nonmetastatic	GC	455	660	OS	25
Gardini 2016	2012–2015	retrospective	Italy	mixed	HCC	56	360	OS, PFS	NR
Huang 2016	2013–2014	retrospective	China	nonmetastatic	GC	445	572	OS	54.6
Gao 2016	2014–2015	retrospective	China	mixed	HCC	183	330	OS	NR
Yu 2016	NR	NR	China	NR	AM	113	615	OS	NR

HCC, hepatocellular carcinoma; MCRPC, metastatic castration-resistant prostate cancer; RCC, renal cell cancer; ESCC, esophageal squamous cell carcinoma; BTC, biliary tract cancer; MOJ, malignant obstructive jaundice; SCLC, small cell lung cancer ; CRC, colorectal cancer; GC, gastric cancer; AM, acral melanoma; OS, overall survival; PFS, progression-free survival; CSS, cancer-specific survival; NR, not reported.

### Overall survival

**Figure 2:**
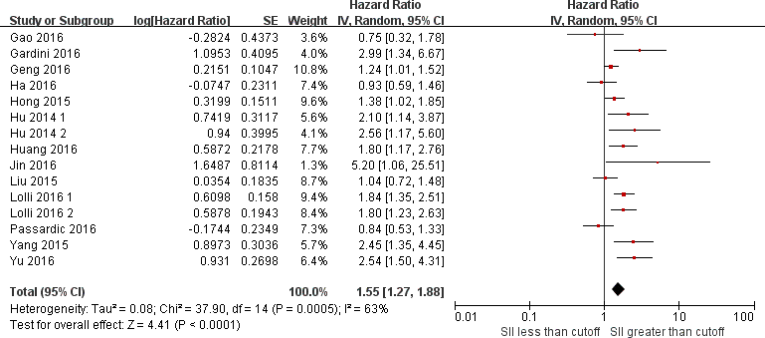
Forest plots of studies evaluating the association between SII and overall survival The center of each square represents the HR, the area of the square is the number of sample and thus the weight used in the meta-analysis, and the horizontal line indicates the 95% CI.

**Figure 3:**
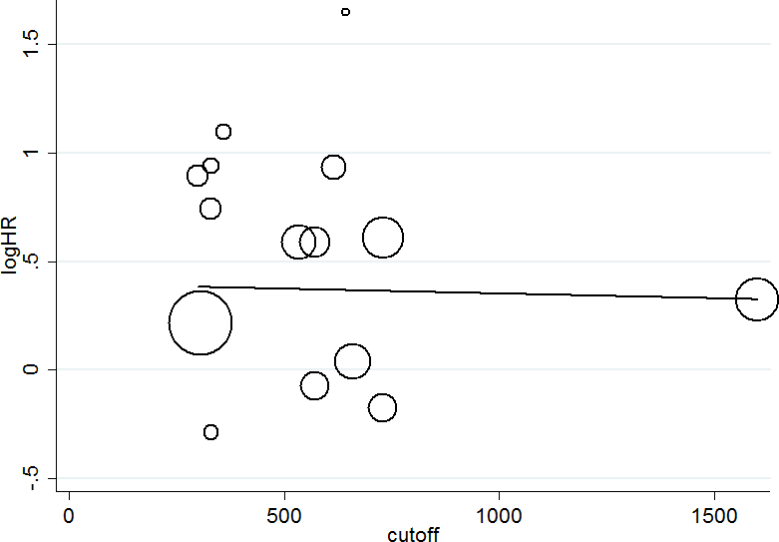
Univariate meta-regression exploring the association of the cutoff used to define SII and the hazard ratio for overall survival

**Figure 4:**
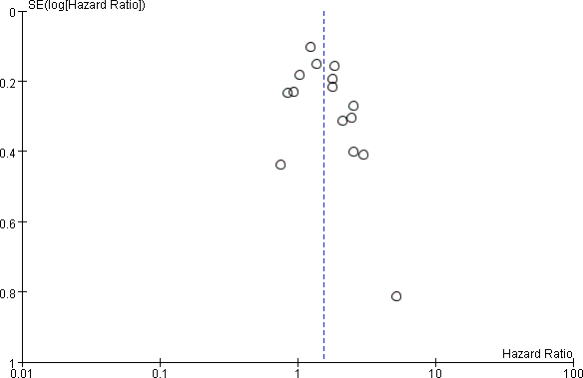
Funnel plots of hazard ratio for overall survival for high SII (horizontal axis) and the standard error (SE) for the hazard ratio (vertical axis) Each study is represented by one circle. The vertical line represents the pooled effect estimate.

15 studies including 4577 patients reported HR for OS. The median cutoff for high SII was 572 (range = 300–1600). SII greater than the cutoff predicted poor OS (HR = 1.55, 95% CI = 1.27–1.88; *P <* 0.001, Figure 2). However, this result is with significant between-study heterogeneity (I-square = 63%, *P <* 0.001). The influence of SII on OS among cancer subgroups is shown in Table 2. High SII was associated with significantly worse OS for acral melanoma, hepatocellular carcinoma, urinary cancers, small cell lung cancer, gastrointestinal tract cancers (HRs = 2.54, 2.08, 1.82, 1.38, 1.21, respectively). The influence of SII on OS among different cancer stages is shown in Table 2. The HRs were 1.65 (95% CI = 1.28–2.13) for metastatic/mixed diseases, 1.48 (95% CI = 1.06–2.05) for nonmetastatic diseases. The subgroups analyses according to cutoffs for SII, data collection, analysis of HR, article type regarding the effect of SII on OS is shown in Table 2. Meta-regression scatter plot is shown in Figure 3. Overall, there was no association between SII cutoff and the HR for OS (*P =* 0.519). Sensitivity analyses investigating the influence by omitting one study at a time and calculating the combined HRs. Any single study did not substantially affect the pooled HRs when deleted from the whole study. Begg’s funnel plot and Egger’s test were conducted to estimate the publication bias of studies. There was no publication bias because of bias exploration funnel plots demonstrated symmetry (Figure 4). Egger’s test also validates little publication bias (*P =* 0.123).

**Table 2:** Subgroup analyses of overall survival

**Subgroup**	**Number of studies**	**HR (95% CI)**	***P***	***P_h_***	***P* for subgroup difference**
Disease site					0.001
Gastrointestinal tract cancers	4	1.21 (1.03–1.41)	0.02	0.09	
Urinary cancers	2	1.82 (1.43–2.32)	< 0.001	0.93	
Hepatocellular carcinoma	5	2.08 (1.52–2.85)	< 0.001	0.15	
Small cell lung cancer	1	1.38 (1.02–1.85)	0.03	-	
Acral melanoma	1	2.54 (1.50–4.31)	< 0.001	-	
Other	2	1.84 (0.35–9.63)	0.47	0.04	
Disease stage					0.60
Metastatic/mixed	9	1.65 (1.28–2.13)	0.0001	0.01	
Nonmetastatic	4	1.48 (1.06–2.05)	0.02	0.02	
Cutoffs for SII					0.31
≥ 572	8	1.42 (1.08–1.87)	0.01	0.002	
< 572	7	1.76 (1.29–2.41)	< 0.001	0.02	
Analysis of HR					0.10
Multivariable	14	1.59 (1.31–1.93)	< 0.001	< 0.001	
Univariable	1	0.75 (0.32–1.78)	0.52	-	
Data collection					0.02
Prospective	1	0.84 (0.53–1.33)	0.46	-	
Retrospective	13	1.56 (1.28–1.89)	< 0.001	0.005	
Article type					0.06
Abstract	1	2.54 (1.50–4.31)	< 0.001	-	
Full paper	14	1.49 (1.23–1.81)	< 0.001	0.002	

The subgroup “gastrointestinal tract cancers” includes 1 esophageal squamous cell carcinoma studies, 1 colorectal cancer study and 2 gastric cancer studies; The subgroup “urinary cancers” includes 1 renal cell cancer study and 1 metastatic castration-resistant prostate cancer study; The subgroup “other” includes 1 malignant obstructive jaundice study and 1 biliary tract cancer study. *P*_*h*_, *P*-values for heterogeneity from *Q* test.

### Progression-free survival

Three studies including 680 patients reported HRs for PFS. Overall, SII over the cutoff was not associated with a hazard ratio for worse PFS outcome. The HR for PFS was 1.31 (95% CI = 0.76–2.27, *P =* 0.33, Table 3).

**Table 3:** Subgroup analyses of secondary outcomes

**Secondary outcomes**	**Num of studies**	**HR((95% CI)**	***P***	***P_h_***
PFS	3	1.31 (0.76–2.27)	0.33	0.005
CSS	1	1.44 (1.04–1.99)	0.03	-

PFS, progression-free survival; CSS, cancer-specific survival; *P*h, *P*-values for heterogeneity from *Q* test.

### Cancer-specific survival

Only one study comprising 298 patients reported hazard ratios for CSS. Overall, SII over the cutoff was associated with a hazard ratio for worse CSS outcome. The HR for CSS was 1.44 (95% CI = 1.04–1.99, *P =* 0.03, Table 3).

## DISCUSSION

Many recent studies [8, 9, 16, 20–22, 24, 25, 27, 28] have suggested that an elevated SII is associated with poor overall survival or progression free survival of patients with cancers. While several studies [18, 19, 23, 26] show that SII is not significantly associated with overall survival. Thus the results of published studies are inconsistent. Here we undertook a meta-analysis of 16 studies including 4875 patients with solid tumors to assess whether SII was associated with prognosis of solid tumor. Overall, we found a significant association between elevated SII and poor survival. Consistent results were observed in various cancer subgroups, including hepatocellular carcinoma, gastrointestinal tract cancers, urinary cancers, small cell lung cancer and acral melanoma. Differences in HRs were observed between cancer sites and may be the result of inflammation playing different roles in different types of cancer. In addition, a trend for the association of high SII with worse OS was greater for metastatic/mixed cases than that in the nonmetastatic diseases. This may reflect either increased tumor burden or a long term inflammatory process [29]. The cutoff of SII in included studies didn’t reach a standard point, and the method to determine the cutoffs is not described in many studies. Cutoffs of SII varied among the included studies, however, meta-regression analysis showed that there was no association between SII cutoff and reported HR for OS. Thus, it was unlikely to influence our results. However, the cutoff value must be established in one cohort of patients and tested in another and the number of patients in each group needs to be considered in the statistical analysis [30].

Several studies may give explanations for the prognostic values of SII in tumors: Neutrophils activate endothelium and parenchymal cells via soluble factors secretion, enhancing circulating tumor cell adhesion in distant sites [31–33]. In addition, clinical studies have suggested that increased numbers of circulating neutrophils are associated with adverse prognosis in patients with cancers [34]. The effects of platelets on metastatic have been attributed to their ability to promote adhesion or to their capacity to prevent cell death. This is owing to the physical shield around tumor cells they formed [35]. Therefore, platelets may contribute to progression and metastasis of cancers. Lymphocytes play a crucial role in cancer immune surveillance and defense via cytotoxic cell death and inhibition tumor cell proliferation and migration [4]. Thus, due to high levels of neutrophils and platelets while low level of lymphocytes, a higher SII usually indicates a stronger inflammatory and a weaker immune response in patients. It may be associated with invasion and metastasis of tumor cells and hence lead to poor survival.

Some limitations of our meta-analysis existed. Firstly, the number of published studies included was not large enough for subgroup analysis. Secondly, only studies reporting HR and 95% confidence interval was included in our meta-analysis. Thus, further bias was potentially introduced. In addition, one study only reported univariate hazard ratio, which could introduce a bias toward overestimation of the prognostic role of SII. Finally, lymphocyte, neutrophil and platelet counts are commonly affected by the existence of infection, chemotherapy and other related factors and studies included in this meta-analysis did not strictly control these confounding factors. However, most cases included in the studies are patients who are going to have surgery and chemotherapy, which indicates that most cases were under non-infectious state when the peripheral blood for calculating SII was gathered. But we should admit that there are so many other factors such as the existence of dehydration, which is common but hard to control that affect the SII result.

In conclusion, our meta-analysis suggests elevated SII indicated poor prognosis and SII may serve as a cost-effective prognostic biomarker. Subgroup analyses by cancer sites identified consistent results in various solid tumors, including hepatocellular carcinoma, gastrointestinal tract cancers, urinary cancers, small cell lung cancer and acral melanoma. The clinical significance of SII as a prognostic indicator must be further validated.

## SUPPLEMENTARY MATERIALS FIGURE


