# Supplementary material for: Prognostic role of systemic immune-inflammation index in solid tumors: a systematic review and meta-analysis
Source: Oncotarget. 2017 Jun 29;8(43):75381–8. doi: 10.18632/oncotarget.18856 (PMC5650428; doi:10.18632/oncotarget.18856)
Supplement: Supplementary file 1 [file oncotarget-08-75381-s001.pdf]

Prognostic role of systemic immune-inflammation index in solid tumors: a systematic review and meta-analysis

Supplementary Materials

Supplementary Table 1: Newcastle-ottawa quality assessment scale for cohort studies

Study	Selection (0–4)				Comparability (0–2)		Outcomes (0–3)			Total
	REC	SNEC	AE	DO	SC	AF	AO	FU	AFU	
Hu 2014 1	*	*	*		*		*	*		6
Hu 2014 2	*	*	*		*		*	*		6
Lolli 2016 1	*	*	*		*		*	*		6
Lolli 2016 2	*	*	*		*		*	*		6
Yang 2015	*	*	*		*		*	*		6
Passardic 2016	*	*	*	*	*		*	*		7
Feng 2016	*	*	*		*		*	*		6
Ha 2016	*	*	*		*		*	*		6
Geng 2016	*	*	*		*		*	*		6
Jin 2016	*	*	*		*		*	*		6
Hong 2015	*	*	*		*		*	*		6
Liu 2015	*	*	*		*		*	*		6
Gardini 2016	*	*	*		*		*	*		6
Huang 2016	*	*	*		*		*	*		6
Gao 2016	*	*	*				*	*		5
Yu 2016	-	-	-	-	-	-	-	-	-	-

REC = representativeness of the exposed cohort; SNEC = selection of the non-exposed cohort; AE = ascertainment of exposure; DO = demonstration that outcome of interest was not present at start of study; SC = study controls for age, sex, marital status; AF = study controls for any additional factors; AO = assessment of outcome; FU = follow-up long enough for outcomes to occur; AFU = adequacy of follow-up of cohorts. *Asterisk means that the study is satisfied the item, no asterisk means the opposite situation, and – means the item cannot be assessed in the study.
